# Low-Dose Levodopa Protects Nerve Cells from Oxidative Stress and Up-Regulates Expression of pCREB and CD39

**DOI:** 10.1371/journal.pone.0095387

**Published:** 2014-04-17

**Authors:** Shi-Ying Zhong, Yong-Xing Chen, Min Fang, Xiao-Long Zhu, Yan-Xin Zhao, Xue-Yuan Liu

**Affiliations:** Department of Neurology, Shanghai Tenth People’s Hospital of Tongji University, School of Medicine, Shanghai, China; Indian Institute of Toxicology Reserach, India

## Abstract

**Objective:**

This study aimed to investigate the influence of low-dose levodopa (L-DOPA) on neuronal cell death under oxidative stress.

**Methods:**

PC12 cells were treated with L-DOPA at different concentrations. We detected the L-DOPA induced reactive oxygen species (ROS). Meanwhile, MTT and LDH assay were performed to determine the proliferation and growth of PC12 cells with or without ROS scavenger. In addition, after pretreatment with L-DOPA at different concentrations alone or in combination with CD39 inhibitor, PC12 cells were incubated with hydrogen peroxide (H_2_O_2_) and the cell viability was evaluated by MTT and LDH assay. In addition, the expression of pCREB and CD39 was detected by immunofluorescence staining and Western blot assay in both cells and rat’s brain after L-DOPA treatment.

**Results:**

After treatment with L-DOPA for 3 days, the cell proliferation and growth were promoted when the L-DOPA concentration was <30 µM, while cell proliferation was comparable to that in control group when the L-DOPA concentration was >30 µM. Low dose L-DOPA could protect the PC12 cells from H_2_O_2_ induced oxidative stress, which was compromised by CD39 inhibitor. In addition, the expression of CD39 and pCREB increased in both PC12 cells and rats’ brain after L-DOPA treatment.

**Conclusions:**

L-DOPA at different concentrations has distinct influence on proliferation and growth of PC12 cells, and low dose (<30 µM) L-DOPA protects PC12 cells against oxidative stress which might be related to the up-regulation of CD39 and pCREB expression.

## Introduction

Levodopa (L-DOPA) is the most widely used drug in the treatment of Parkinson’s disease (PD). However, whether L-DOPA has neurotoxicity or not is still unclear. The clinical evidence for its neurotoxicity is based on the fact that L-DOPA alone can not alleviate PD, the honeymoon of this treatment last only 2–5 years and it may finally cause adverse effects in the long term treatment. Furthermore, elevated oxidative stress during the L-DOPA treatment may lead to the gradual degeneration of dopaminergic neurons because the self-oxidation or enzymatic oxidation of L-DOPA may result in the production of reactive oxygen species (ROS), causing damage to neurons, including the residual nigrostriatal dopaminergic neurons [1]. Oxidative stress has been found to involve in the pathogenesis of PD, and L-DOPA may further deteriorate the oxidative stress of the nervous system, leading to the aggravation of PD [2].

However, there is still evidence indicating that L-DOPA has no neurotoxicity. For example, neurons may survive for a few days in the presence of low dose L-DOPA [3]–[6]. *In vivo* study showed that long-term treatment of high dose L-DOPA did not cause damage to dopaminergic cells, while increased the density of dopaminergic nerve fibers [7]–[9]. In patients whose nigra-striatal system is intact, long-term L-DOPA treatment does not cause any damage to the dopaminergic neurons [10]. Recently, a multicenter, randomized, double-blind four-year clinical trial (ELLDOPA) reported the protective effect of L-DOPA in 361 patients with PD [10].

Transcription factors (including cAMP response element binding protein [CREB] family) are closely related to the metabolism of monoamine neurotransmitters including dopamine. After being phosphorylated at amino acid residue Ser133 [11], pCREB binds to the cAMP response element (CRE) and subsequently activates the transcription of downstream genes, playing an important role in the survival and repair of neurons under stress [12]. CREB is regulated by a variety of signaling pathways [13], [14]. Stress including ischemia and hypoxia can phosphorylate CREB and up-regulate the expression of some factors including brain derived neurotrophic factor (BDNF) [15], [16]. Catecholamines such as dopamine (DA) can bind to DA D1 receptor and activate adenylate cyclase – protein kinase A (AC-PKA) signal pathway through the Gs protein, which leads to the phosphorylation of its substrates such as CREB, resulting in increase in pCREB expression [17].

Elevated extracellular ATP is known as a sign of physical stress and cell damage, while adenosine may limit the damage induced by physical defensive response. CD39, a protein expressed on cell surface, plays a neuroprotective role by regulating the T terminal phosphate hydrolysis of ATP and ADP and, together with CD73, turning AMP into adenosine [18]. Although CD39 plays an important role under the stress condition, the regulation of CD39 expression at molecular level is still poorly understood. A silicon analysis shows that there are several CRE-like sequences at the potential regulatory sites of CD39 promoter, one of which is close enough to the transcription start point [19]. Liao et al confirmed that CD39 transcription was regulated through the cAMP-PKA-pCREB pathway [20].

Metabolism of L-DOPA causes oxidative stress in dopaminergic neurons, but a large number of experiments and clinical studies show the neuroprotective effects of L-DOPA. Whether L-DOPA exerts neuroprotective effect via the CAMP-CREB-pCREB-CD39 pathway to alleviate oxidative stress is still unclear. In this study, the protective effect of L-DOPA on PC12 cells against oxidative stress was investigated, and the role of pCREB and CD39 in this protective effect was also explored.

## Materials and Methods

### Ethics Statement

Animal experiments were performed in accordance with the National Guidelines for the Use and Care of Laboratory Animals and this study was approved by the Institutional Animal Care and Use Committee of Tongji University.

### Cell Culture

PC12 cells were from the laboratory of Tenth People’s Hospital of Tongji University and cultured in high glucose DMEM containing 10% horse serum, 5% fetal calf serum (FCS) and penicillin/streptomycin (25 units/ml and 25 µg/ml, respectively) in an atmosphere with 5% CO_2_ at 37°C. Cells were passaged once every 3 days. Further experiments were performed using PC12 cells in the logarithmic growth phase.

### Proliferation of PC12 Cells Treated by L-DOPA at Different Concentrations with or without ROS Scavenger

After passaging, PC12 cells were seeded into 96-well plates (5×10^3^/well; 200 µl/well) and incubated in an environment with 5% CO_2_ at 37°C for 24 h. Then, these cells were divided into 8 groups according to concentrations of L-DOPA: 0, 1, 5, 10, 20 30, 40 and 50 µmol/L [3]–[5]. There were 10 wells in each group. The proliferation and growth of PC12 cells were determined by MTT assay and LDH assay and were observed under an optical microscope after treatment for 3 days. Intracellular ROS levels were measured in each group. In addition, N-acetyl-L-cysteine (NAC; Sigma USA) was used as a ROS scavenger. Cells were pretreated with 10 mM NAC (Sigma, USA) for 24 h and then incubated with L-DOPA at different concentrations. The cell growth was also determined by MTT and LDH assay.

### Viability of L-DOPA Treated PC12 Cells with or without CD39 Inhibitor Pre-treatment Under Oxidative Stress

After passaging, PC12 cells were seeded into 96-well plates (5×10^3^/well; 200 µl/well) and incubated in an environment with 5% CO_2_ at 37°C for 24 h. These cells were divided into two groups: L-DOPA treatment group and CD39 inhibitor pre-treatment group (cells were pre-incubated with 0.5 mM ARL 67156 [6-N, N’-diethyl-D-β-γ-dibromomethylene ATP, Santa Cruz USA] for 30 min or 10 µM H89 [a PKA inhibitor] for 1 h before L-DOPA treatment). The nucleotide analogue ARL 67156 is a selective inhibitor of ecto-ATPase of CD39/NTPDase family [69]. Cells were then treated with L-DOPA at different concentrations (0, 1, 5, 10 and 20 µmol/L) for 3 days. On the fourth day, cells were incubated with 100 µmol/L H_2_O_2_ for 8 h. Finally, MTT assay and LDH assay were performed to determine the viability of PC12 cells. The oxidative stress induced by H2O2 was evaluated by detection of ROS.

### Methylthiazol Tetrazolium (MTT) Assay

In brief, culture medium was removed and cells were washed with PBS twice before they were incubated with 20 µL MTT (5 mg/mL, Sigma USA) for 4 h at 37°C. Formazan crystals in the cells were solubilized using dimethyl sulfoxide with plate shaking for 10 min. Absorbance (A) was measured at 570 nm with a reference wavelength of 655 nm. The OD of PC12 cells treated with L-DOPA at different concentrations was normalized to that of untreated cells. Cell proliferation (%) = (A_sample_ − A_blank_)/(A_control_ − A_blank_)×100%. (A_sample:_ average OD of L-DOPA-treated cells, A_control_: average OD of L-DOPA-untreated cells, A_blank_: MTT+medium).

### Lactate Dehydrogenase (LDH) Assay

LDH was detected with a diagnostic kit (Jiancheng Bioengineering, Nanjing, China) according to the manufacturer’s instructions. Briefly, the supernatant from each well was collected, and 0.2% Triton X-100 (Sigma, St. Louis, MO, USA) was added to each well to lyse cells. The lysate was centrifuged at 10,000×g for 2 min at 4°C and the supernatant was collected. Then, the samples were incubated with NADH and pyruvate for 15 min at 37°C, and the reaction was stopped by adding 0.4 M NaOH. The activity of LDH was calculated after detection of A at 440 nm. The percentage of specific LDH release was determined as follow: LDH release (%) = (LDH_supernatant_ − LDH_blank_)/(LDH_supernatant_ + LDH_lysate_ − LDH_blank_)×100%.

### Measurement of Intracellular ROS

The intracellular ROS was estimated using the ROS Fluorometric assay kit (GENMED SCIENTIFICS INC. USA) following the manufacturer’s instructions. Briefly, 100 µl of staining fluid containing reagent B and reagent C was added into each well after the medium was removed. After incubation at 37°C, the fluid was replaced with 100 µl of reagent D. Then, these cells were examined with a fluorescence microplate reader.

### Immunofluorescence Staining

PC12 cells were placed on coverslips in 6-well plates (8×10^6^/well; 3 ml/well) and incubated in an environment with 5% CO_2_ at 37°C for 24 h. Then, these cells were divided into 4 groups according to the concentrations of L-DOPA: 0, 1, 5, 10 and 20 µmol/L. After treatment for 3 days, cells were processed as follows: cells were washed in 1× PBS, and fixed for 20 min in 4% paraformaldehyde. Then, cells were treated with 0.2% Triton and 1% BSA for 1 h and with anti-CD39 or anti-pCREB antibody (1∶400, Abcam) for 45 min. Following incubation with fluorescein isothiocyanate-conjugated rabbit anti-rat IgG (1∶1000, Abcam) and DAPl (1∶5000) for 30 min at room temperature, mounting was performed with antifade reagents (Prolong Gold), and photomicrographs were captured using an Olympus microscope.

### Total Protein Extraction

Total protein lysate was prepared using RIPA buffer (50 mM Tris, 150 mM NaCl, 1% Triton X-100, 1% sodium deoxycholate, 0.1% SDS, pH 7.4) in the presence of proteinase and phosphatase inhibitor cocktail (Sigma, MO, USA). For protein extraction, PC12 cells were transferred into 150-mm dishes (1.5×10^6^/dish; 8 ml/dish) and incubated in an environment with 5% CO_2_ at 37°C for 24 h. Then, these cells were divided into 4 groups according to the concentrations of L-DOPA: 0, 1, 5, 10 and 20 µmol/L. After treatment for 3 days, these cells were washed twice with cold PBS and lysed in 300 µL of RIPA buffer. Then, these cells were transferred into eppendorf tubes and incubated on ice for 30 min, followed by vortexing once every 10 min. The supernatant was collected after centrifugation at 12000 rpm for 5 min at 4°C. Protein concentrations were determined by BCA protein assay. Loading buffer was added into each sample and the mixture was stored at −80°C after boiling for 10 min.

### Western Blot Assay

Samples (20 µg protein) were subjected to 10% SDS-polyacrylamide gel electrophoresis and electrophoretically transferred to Immun-Blot polyvinylidene difluoride membranes (Millipore, Bedford, MA, USA). After blocking in Tris-buffered saline in 0.05% Tween-20 (TBST) containing 5% nonfat dry milk for 1 h, the membranes were washed thrice with TBST at room temperature and then incubated with primary antibody in 3% BSA/PBS overnight (anti-CD39: abcam 1∶2000; anti-pCREB: abcam 1∶1500; tubulin: 1∶2000) at 4°C. Incubation was done with with the secondary antibody (rat anti-rabbit 1∶5000; Abcam, Britain) at room temperature for 1 h. The protein bands were scanned with the Odyssey color infrared fluorescence imaging system (LI-COR Company, USA). The expression of target proteins was normalized to that of β-actin.

### Animals

In this study, 18 male Sprague Dawley (SD) rats weighing 200–250 g were purchased from the Animal Center of Tongji University, Shanghai, China. Animals were allowed to acclimatize to the environment for 7 days before experiments. These rats were housed in an air-conditioned room at a constant temperature of 22±1°C with 12∶12 h light/dark cycle and given *ad libitum* access to water and food.

### Pharmacological Treatments

The animals were randomly divided into 4 groups (n = 6 per group) in which rats were treated with L-DOPA at 120 mg/kg (highest dose group, HstG), 60 mg/kg (high dose group, HG), 30 mg/kg (low dose group, LG), and 0 mg/kg (control group, CG). L-DOPA was administered intraperitoneally (i.p.) in HstG, HG and LG for 7 days. In CG group, 0.1 M HCL was administered once daily for 7 days. At the end of study, animals were sacrificed by over dose of anesthesia (30 mg/kg sodium pentobarbital). The brain was harvested for biochemical examinations.

### Immunofluorescence Staining and Western Blot Assay

For immunofluorescence staining, animals were intraperitoneally anesthetized with sodium pentobarbital (30 mg/kg) and perfused transcardially with 150 mL of PBS and then with 400 mL of ice-cold 4% paraformaldehyde in phosphate buffer (PB; pH 7.4). The brain was quickly harvested and post-fixed for 24 h in 4% paraformaldehyde. The 4-mm coronal slices containing cortex and substantia nigra were obtained. Paraffin-embedded sections (4 µm in thickness) were incubated with primary antibodies against anti-CD39 or anti-PCREB (1∶200 Abcam) at 4°C overnight and then with fluorescein isothiocyanate-conjugated rabbit anti-rat IgG. Images were captured using an Olympus microscope (Center Valley, PA), and Image-Pro Plus software 6.0 (USA) was used to detect the fluorescence intensity. Six sections per rat were visualized, and positively stained cells were counted in five randomly selected fields (magnification: ×200). The number of positive cells in 30 fields per rat (n = 6 per group) was averaged.

For Western blot assay, animals were intraperitoneally anesthetized with sodium pentobarbital at 30 mg/kg and the rat brain was harvested. Then, the striatal tissues were collected, frozen rapidly, homogenized, subjected to 10% SDS-polyacrylamide gel electrophoresis and then transferred onto Immun-Blot polyvinylidene difluoride membranes (Millipore, Bedford, MA, USA).

### Statistical Analysis

Immunofluorescence Images were viewed and captured using an Olympus microscope, and the Image-Pro Plus software 6.0 was used to analyze the immunofluorescence intensity. The product of intensity and area of protein bands represents the relative protein expression. Statistical analysis was performed with SPSS version 18.0. Data were expressed as mean ± standard deviation. One-way analysis of variance (ANOVA) and paired student’s t-test were used for comparisons and data were from at least three independent experiments. A value of P<0.05 was considered statistically significant.

## Results

### Influence of L-DOPA at Different Concentrations on the Growth of PC12 Cells is Related to the ROS Due to L-DOPA Metabolism

**Figure 1:**
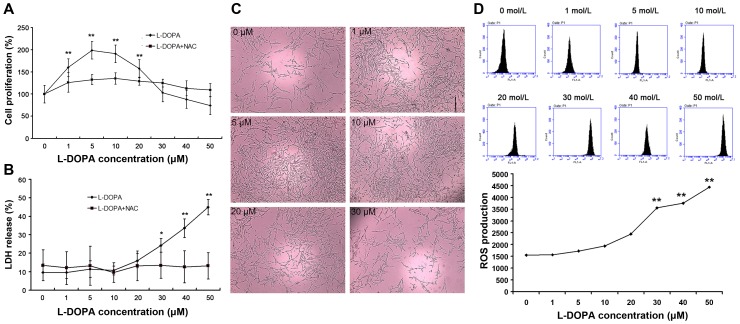
Effect of L-DOPA at different concentrations on proliferation of PC12 cells with or without ROS scavenger (χ±σ, n = 10). PC12 cells were treated with 0, 1, 5, 10, 20, 30, 40 and 50 µmol/L L-DOPA for 3 days with or without NAC (as ROS scavenger) pretreatment. The cell proliferation and growth were detected by MTT assay and LDH assay and cells were also observed under an optical microscope. The OD in treated groups was compared with that of untreated group. (A) The cell proliferation with or without NAC pretreatment was detected by MTT. Results showed L-DOPA at lower than 30 µM promoted the proliferation of PC12 cells, while proliferation remained unchanged when L-DOPA was greater than 30 µM. This promoted proliferation was compromised by NAC. (B) The cell growth was detected by LDH assay. Results showed that the LDH content in 1, 5, 10 and 20 µM groups was lower than that in 30, 40 and 50 µM groups, meaning that cells had better growth in lower L-DOPA concentration groups. NAC could also eliminate this influence. (C) The growth of PC12 cells without NAC pretreatment was observed under an optical microscope (×100). PC12 cells had a higher intensity after treatment with L-DOPA at 1, 5, 10 and 20 µM, while the number of PC12 cells reduced in 30, 40 and 50 µM groups. (D) Intracellular ROS level was measured in each group. The higher the L-DOPA concentration, the higher the ROS level was. *P<0.01, **P<0.05 vs. 0 µmol/L L-DOPA group.

PC12 cells were treated with L-DOPA at different concentrations for 3 days, and the cell proliferation and growth were detected by MTT assay and LDH assay (Figure 1A, B). MTT assay showed the proportion of viable cells was 159.85±37.3% (P<0.01), 198.91±38.1% (P<0.01), 191.07±31.9% (P<0.01), 158.03±28.3% (P<0.01) 103.03±28.3% (P>0.05), 88.00±18.4% (P>0.05) and 74.13±21.1% (P>0.05), and LDH assay showed that was 9.68±4.52% (P>0.05), 9.62±3.27% (P>0.05), 11.44±4.44% (P>0.05), 15.98±3.85% (P>0.05), 24.25±3.71% (P<0.05), 33.59±5.09% (P<0.01) and 44.89±4.16% (P<0.01) after treatment with L-DOPA at different concentrations. L-DOPA at lower than 30 µM promoted the growth of PC12 cells and the maximal proliferation was observed when the L-DOPA was 5 and 10 µmol/L, while proliferation remained unchanged when the L-DOPA was greater than 30 µM. These findings were consistent with previously reported [3]–[5]. The change in the growth of these cells was also observed under an optical microscope (Figure 1C). PC12 cells had higher density after treatment with L-DOPA at 1, 5, 10 and 20 µM, while the number of PC12 cells reduced after treatment with L-DOPA at 30 µM. Detection of ROS showed that the higher the concentration of L-DOPA, the higher the ROS level was (Figure 1D). After NAC treatment, even if the L-DOPA was greater than 30 µM, the cell growth was improved obviously and LDH in the supernatant also decreased significantly (Figure 1A, B). Elevated ROS influences the growth of PC12 cells, while the mechanism underlying the promoted growth of PC12 cells at low dose L-DOPA treatment remains to be further elucidated.

### Low-dose L-DOPA Pretreatment Protects PC12 Cells from Oxidative Stress, which is Compromised by CD39 Inhibitor

**Figure 2:**
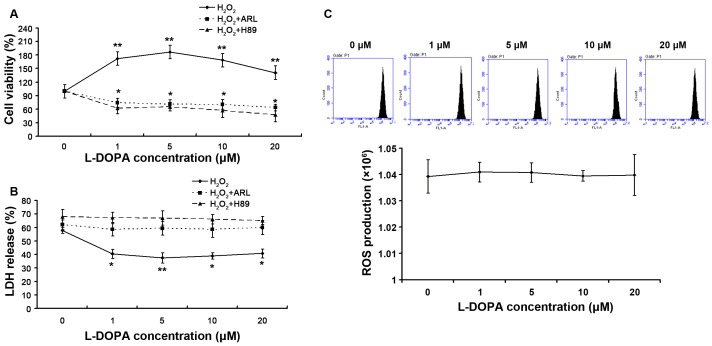
Effect of low dose L-DOPA with or without CD39 inhibitor pre-treatment on the oxidative stress (χ±σ, n = 10). PC12 cells were seeded into 96-well plates (5×10^3^/well; 200 µl/well) and divided into two groups: L-DOPA treatment group and CD39 inhibitor pre-treatment group (cells were pre-incubated with 0.5 mM ARL 67156 for 30 min or 10 µM H89 for 1 h before L-DOPA treatment). Cells were incubated with L-DOPA at different concentrations (0, 1, 5, 10 and 20 µmol/L) for 3 days. On the fourth day, cells were incubated with 100 µmol/L H_2_O_2_ for 8 h. MTT assay and LDH assay were performed to determine the viability of PC12 cells. (A) The cell viability with or without CD39 inhibitor pretreatment was detected by MTT assay. Results showed the viability increased as compared to untreated group, while H89 and ARL eliminated this effect. (B) The cell viability was detected by LDH assay. Results showed that the LDH content in L-DOPA treated groups was lower than that in untreated group, while H89 and ARL eliminated this effect. (C) Intracellular ROS level was measured. H_2_O_2_ could induce oxidative stress. **P<0.01, *P<0.05 vs 0 µmol/L L-DOPA group.

PC12 cells were treated with H_2_O_2_ to induce oxidative stress (Figure 2C) after pretreatment with low dose L-DOPA, and the cell viability was determined by MTT assay and LDH assay. As compared to untreated groups, the viability was 172.41±21.96% (P<0.01), 186.89±30.49% (P<0.01), 169.59±30.28% (P<0.01) and 140.89±23.24% (P<0.01) after L-DOPA pre-treatment (Figure 2A). Meanwhile, LDH content was lower in the L-DOPA pre-treated groups (Figure 2B). In CD39 inhibitor (ARL and H89) pre-treatment group, the viability was 74.22±9.71% (P<0.05), 71.13±10.03% (P<0.05), 70.62±10.89% (P<0.05), 64.10±7.27% (P<0.05) and 62.38±12.64% (P<0.05), 65.69±9.45% (P<0.05), 57.11±15.06% (P<0.05) and 47.71±15.41% (P<0.05), respectively (Figure 2A). Meanwhile, LDH content stood at the same level when compared with control group (Figure 2B). These results confirmed that low dose L-DOPA pretreatment protected PC12 cells from oxidative stress which was compromised by CD39 inhibitor. Thus, the PKA-CD39 pathway may play an important role in the neuroprotection of L-DOPA.

### L-DOPA Increases CD39 and PCREB Expression

**Figure 3:**
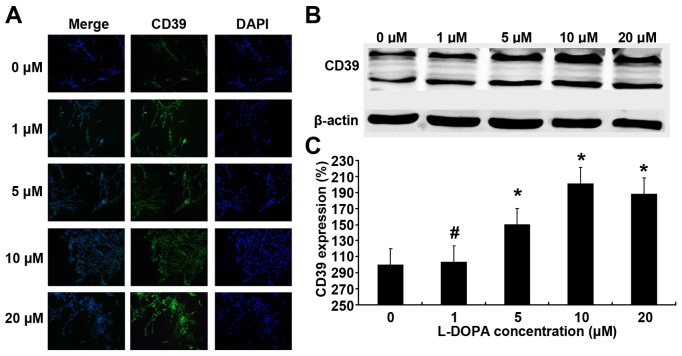
Effect of low dose L-DOPA on CD39 protein expression. PC12 cells were treated with L-DOPA at different concentration for 3 days, and CD39 protein expression was measured by immunofluorescence staining (A) and western blot assay (B). L-DOPA treatment resulted in an increase in CD39 expression when compared with un-treated group and peaked at 10 µM L-DOPA (C). *P<0.05 vs. untreated group. #P>0.05 vs. untreated group.

**Figure 4:**
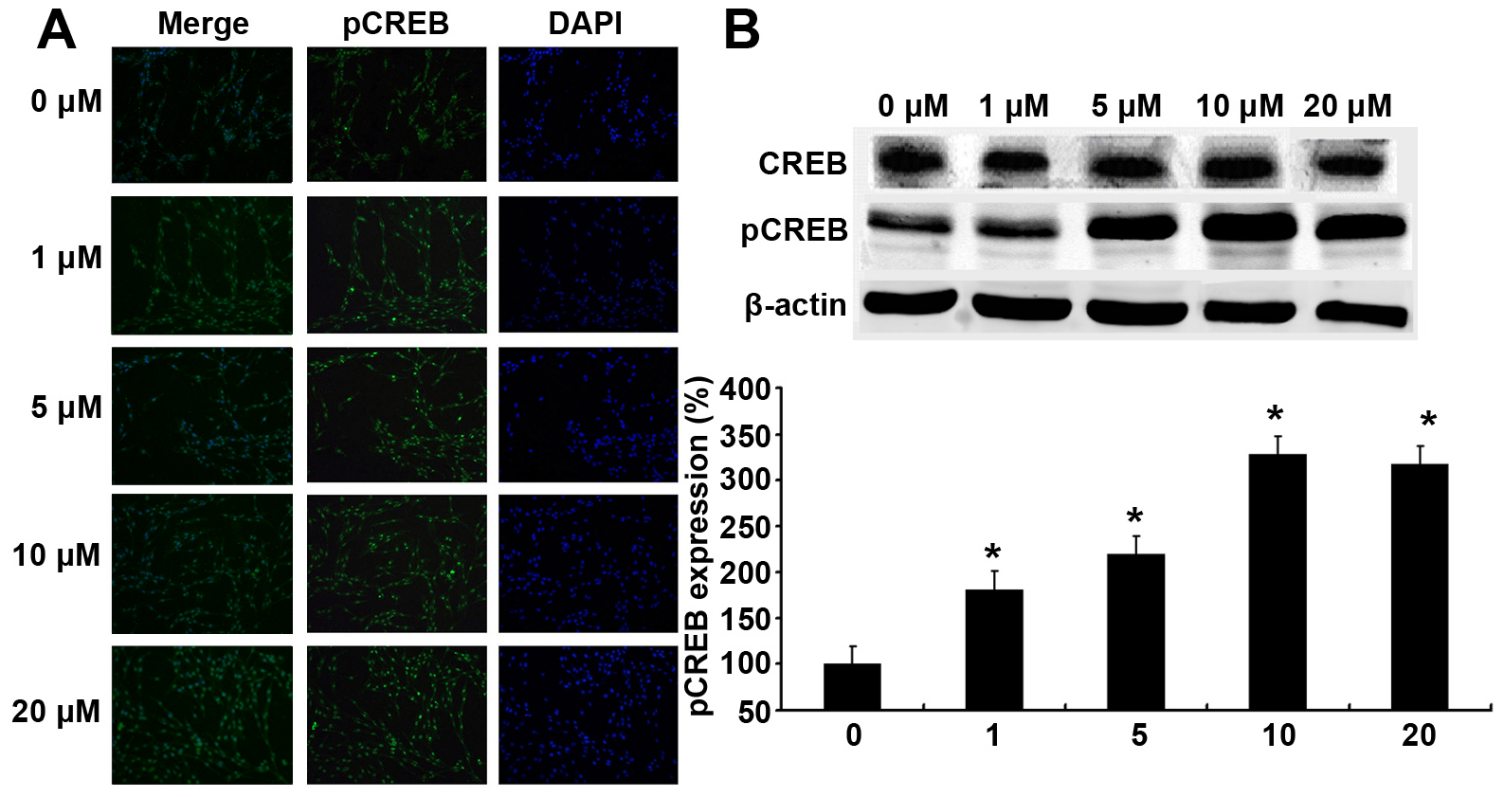
Effect of low-dose L-DOPA on PCREB protein expression. PC12 cells were treated with L-DOPA at different concentrations for 3 days, and PCREB protein expression was measured by immunofluorescence staining (A) and western blot assay (B). L-DOPA treatment increased pCREB expression when compared with untreated cells and peaked at 10 µM L-DOPA (C). *P<0.05 vs. untreated group.

PC12 cells were treated with L-DOPA at different concentrations for 3 days, and the CD39 and PCREB protein expression was measured by immunofluorescence staining and western blot assay. CD39 is a transmembrane protein and pCREB is a nuclear protein. Results showed that fluorescence intensity was higher in the L-DOPA treated groups than in untreated group, showing that CD39 and pCREB expression increased after L-DOPA treatment. (Figure 3A, 4A). Western blot assay showed the same tendency in CD39 and pCREB expression (Figure 3B, 3C, 4B, 4C). The expression of CD39 increased with the increase in the L-DOPA concentration and reached the peak at 10 µM L-DOPA. The level of CREB expression was comparable among groups, while pCREB expression also peaked at 10 µM L-DOPA.

### L-DOPA Increases CD39 and PCREB Expression in the Rat Brain

**Figure 5:**
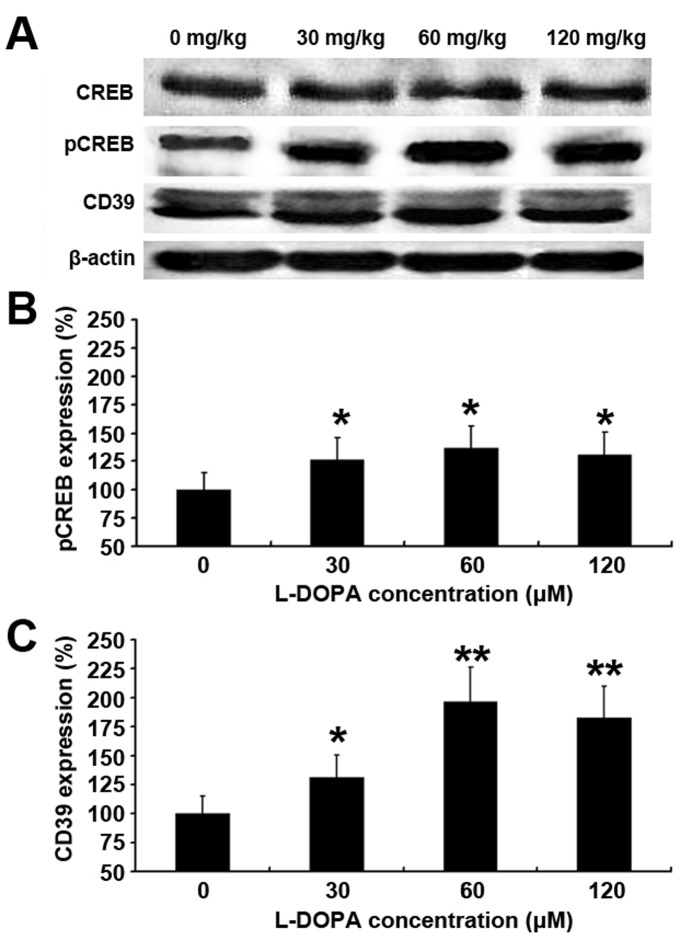
Western blot assay of CD39 and pCREB protein expression in the brains of rats treated with L-DOPA. Rats were treated with L-DOPA at different concentrations (0, 30 60 and 120 mg/kg) once daily for 7 days, and the brains were quickly harvested for western blot assay of the expression of CD39 and pCREB (A). Protein bands of pCREB and CD39 were scanned, and their OD was determined and normalized to that of an internal reference (B, C). *P<0.05 and **P<0.01 vs. CG group.

**Figure 6:**
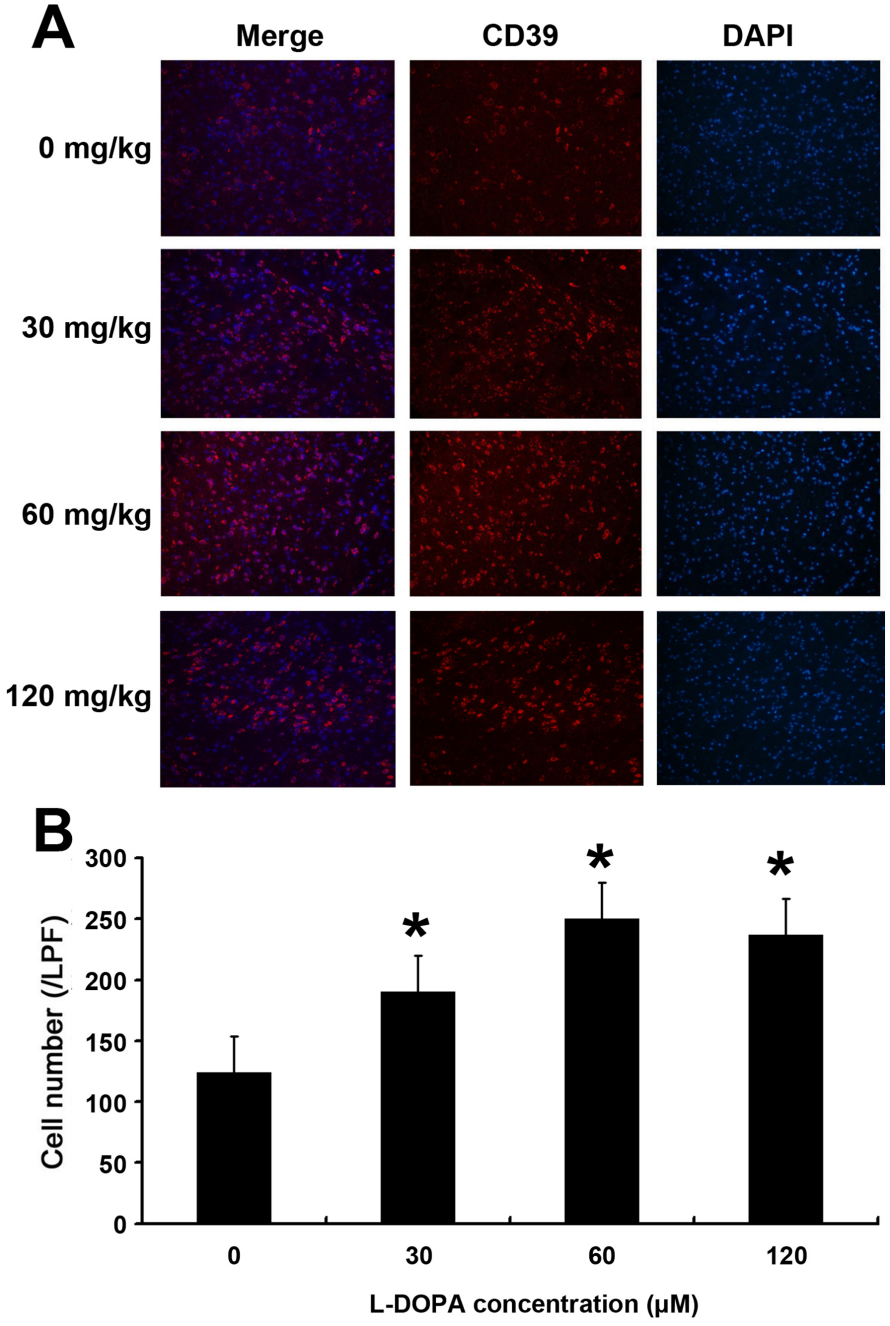
Effect of L-DOPA on CD39 protein expression in the rat brains. Rats were treated with L-DOPA once daily for 7 days. The brains were quickly harvested for immunofluorescence staining of CD39 (A). The number of CD39 positive cells (n = 6 per group) was determined (B). The number of CD39 positive cells in L-DOPA treated groups was significantly larger than that in CG group. *P<0.05 vs. CG group.

**Figure 7:**
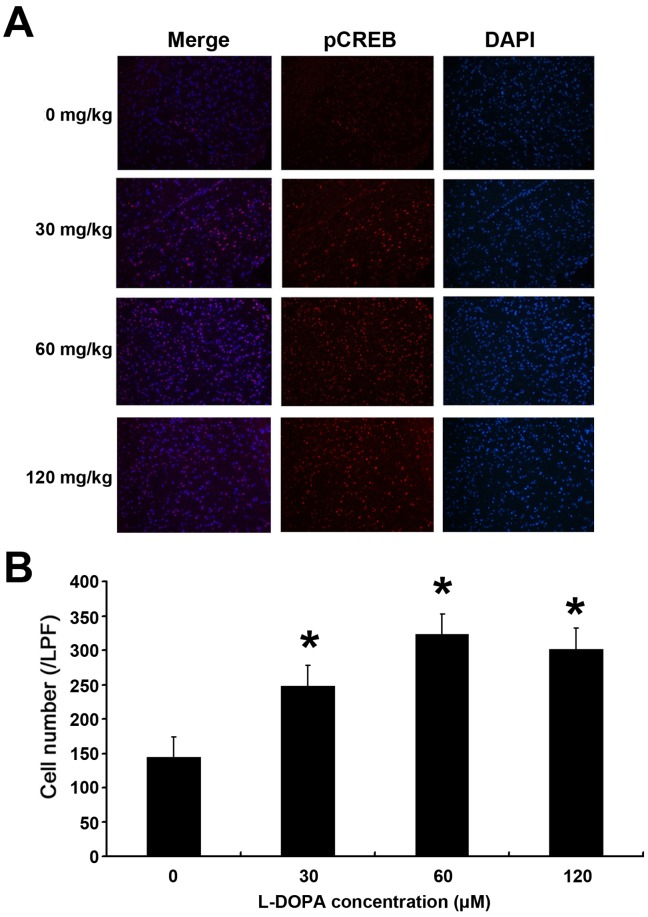
Effect of L-DOPA on pCREB protein expression in the rat brains. Rats were treated with L-DOPA once daily for 7 days and the brains were quickly harvested for immunofluorescence staining of pCREB expression (A). The number of pCREB positive cells (n = 6 per group) was determined (B). The number of pCREB positive cells in L-DOPA treated groups was markedly larger than that in CG group. *P<0.05 vs. CG group.

Rats were intraperitoneally administered with 120 mg/kg L-DOPA in HstG, 60 mg/kg L-DOPA in HG, 30 mg/kg L-DOPA in LG and solvent in CG group once daily for 7 days. The brains were quickly harvested for western blot assay and immunofluorescence staining was performed to determine the expression of PCREB and CD39 (Figure 5, 6, 7). Western blot assay (Fig. 5A) showed pCREB and CD39 expression was greater in L-DOPA treated groups than in CG (126.74±4%, 136.21±5% and 131.14±5%, 131.24±4%, 196.72±6% and 182.85±5%, respectively; P<0.05 vs. CG; n = 6; Fig. 5B, 5C). The number of pCREB and CD 39 positive cells in 30 fields per rat (n = 6 per group) was averaged (Figure 6A, 7A). The number of pCREB and CD39 positive cells in L-DOPA treated groups was markedly larger than that in CG (144±14, 248±10, 323±16 and 302±11; 124±11, 190±13, 250±13 and 237±11, respectively; P<0.01 vs. CG; n = 6; Figure 6B, 7B).

## Discussion

Whether L-DOPA is neurotoxic or neuroprotective is still an issue of debate. The plasma concentration of L-DOPA in PD patients is generally 5–50 µM, and L-DOPA at a physiological low concentration (50 µM) may nourish and protect the neurons [5]. In addition, L-DOPA may enhance the antitoxic capacity of dopaminergic neurons against certain toxics [6]. Studies have shown that L-DOPA at a low concentration may also increase the survival and number of neuritis of fetal rat midbrain neurons and cortical astrocytes [3], [4]. In healthy animals, long-term application of high dose L-DOPA fails to reduce the number of dopaminergic cells, and DA nerve fiber density increases in PD animals [7]–[9]. Patients who have been misdiagnosed with PD (such as essential tremor or vascular parkinsonism patients) may not develop symptoms of PD after long-term use of L-DOPA, PET does not show the change in L-DOPA level and dopaminergic neuronal injury is also not observed in autopsy of these patients [10]. Neuropathology shows patients receiving L-DOPA treatment have not more substantia nigra abnormalities than those without L-DOPA treatment, and the mortality of untreated patients is three times that of healthy control, while that of treated group is 1.5 times [10]. The ELLDOPA study shows high dose L-DOPA does not accelerate the disease progression, but exerts a protective effect in 361 patients with clinically diagnosed PD in early phase (within 2 years of initial diagnosis and absence of pharmacotherapy) [10]. Thus, the effect of L-DOPA on PD still is needed to be investigated in more studies.

In our experiment, the neuroprotective effect of L-DOPA was investigated in the presence of H_2_O_2_ induced oxidative stress. PC12 cells were used because they can metabolize dopamine and have typical neuronal characteristics. PC12 cells were treated with L-DOPA at different concentrations. Results showed that L-DOPA at lower than 30 µM promoted the growth of PC12 cells. To characterize the role of L-DOPA in oxidative stress, the survival of PC12 cells pretreated with low dose L-DOPA was determined after H_2_O_2_ treatment. Results demonstrated that low dose L-DOPA protected nerve cells from oxidative stress via up-regulating pCREB and CD39. We postulate that the damage of ROS produced by high dose L-DOPA metabolism overwhelms the L-DOPA induced protection, and thus the cell growth and survival became worse. CD39 is a 70–100 kD transmembrane protein with two transmembrane domains and a large extracellular region [21]. The extracellular domain of CD39 on the endothelial cells is exposed to the blood flow, and can regulate the purine signal transmission and phosphorylation of ATP and ADP into AMP, further together with CD73 turning AMP into adenosine [18]. Thus, CD39 can metabolize ATP and ADP released by activated platelets to reduce the platelet activation and recruitment. In addition, it maintains the blood flow in the absence of prostacyclin and nitric oxide, reducing the risk for thrombosis. CD39 also activates the type II immune response by regulating the expression of cytokines, inducing cell adhesion [22]–[24] and leukocyte migration [25], which reduces inflammation and regulates the immune function, exerting protective effect on the ischemia-reperfusion injury of the heart and brain [26]–[33]. Human CD39 gene comprises an open reading frame encoding about 510 amino acids, of which there are six N-glycosylation sites, 11 cysteine residues and two transmembrane domains [21]. Although CD39 is expressed on a variety of cells, including endothelial cells, macrophages and lymphocytes, and is very important for the metabolism of ATP and ADP under stress, the regulation of CD39 expression at molecular level is still poorly understood.

Liao et al. reported that cAMP could phosphorylate CREB Ser133 via the PKA signaling pathway to activate the CD39 transcription after pCREB binds to the promoter region of CD39 [20]. Their results showed that CREB plays an important role in not only the transcription of CD39, but also the activity of CD39. cAMP analogue (8-bromo-cAMP) is able to remarkably induce CD39 expression (more than 40 times) in macrophages, and raise the CD39 antigenicity and activity significantly according to the platelet aggregation determination. H89 (PKA inhibitor) reduced the 8-bromo-cAMP-induced mRNA expression of CD39 by 98%, and subsequently the protein expression of CD39 decreased. Interaction among PKA, ERK, and CREB/ATF signaling pathways, independently sometime, may be a mechanism underlying the cAMP induced regulation of CD39 transcription.

Repeated administration of L-DOPA triggers a signaling cascade [34]–[36]. D1-like receptors link to the stimulatory G protein (Gs), which increases the intracellular cAMP. cAMP may induce PKA activation, and in turn the activated PKA phosphorylates the cytoplasmic and nuclear proteins and regulates the ion channel function and gene expression [37]–[39]. Via a PKA-dependent mechanism, stimulation of D1 subtype will activate the cAMP/PKA/p38MAPK/JNK/ERK signaling pathways [40]–[44], playing an important role in the phosphorylation [45]–[49]. The transcriptional activation of CREB depends on its phosphorylation at Ser-133 either directly or indirectly by PKA [16], [50]–[55]. D1 receptors can also influence the CaMK-mediated phosphorylation of CREB [56], [57]. Other studies also found the DA-induced alteration of CREB in organotypic slice cultures [58] and alteration of ERK in a dopaminergic cell line and primary DA neurons from rat substantia nigra [59]. After treated with L-DOPA for 3 weeks (twice, daily), the striatal pCREB protein expression and number of positive neurons increased. Intrastriatal administration of CREB antisense or PKA inhibitor Rp-cAMPS was also found to attenuate the L-DOPA-induced pCREB elevation [60]. Chronic L-DOPA treatment results an increase in the striatal pCREB [57], [61]–[63]. Augmented pCREB expression during chronic L-DOPA treatment is mainly found in the dorsolateral striatum and thus most likely to affect the striatal dopaminergic neurons [64], [65]. Other studies concluded that neurons under stress might activate CREB phosphorylation as a protective response [66]. Taken together, above findings indicate that L-DOPA-induced D1 DA receptors activation is an important trigger of MAPK signaling and CREB phosphorylation in the striatal neurons, which plays an important role in promoting neuronal survival and synaptic plasticity [51], [67], [68].

In general, L-DOPA is able to promote the binding of D1-like receptors to Gs which increases the intracellular cAMP. Under oxidative stress, self-oxidation and enzymatic oxidation of L-DOPA generates ROS, which increases cAMP to protect against oxidative stress. Through cAMP-PKA pathway, CREB (Ser133) phosphorylation increases and the binding of pCREB to CRE of CD39 promoter is enhanced, where the phosphorylated Ser133 works as a bracket for the transcription of CREB-binding protein and its homologous gene P300 to start the gene transcription.
